# Continuous exposure to *Plasmodium *results in decreased susceptibility and transcriptomic divergence of the *Anopheles gambiae *immune system

**DOI:** 10.1186/1471-2164-8-451

**Published:** 2007-12-05

**Authors:** Ruth Aguilar, Suchismita Das, Yuemei Dong, George Dimopoulos

**Affiliations:** 1 W. Harry Feinstone Department of Molecular Microbiology and Immunology, Bloomberg School of Public Health, Johns Hopkins University, 615N. Wolfe Street, Baltimore, MD 21205-2179, USA

## Abstract

**Background:**

*Plasmodium *infection has been shown to compromise the fitness of the mosquito vector, reducing its fecundity and longevity. However, from an evolutionary perspective, the impact of *Plasmodium *infection as a selective pressure on the mosquito is largely unknown.

**Results:**

In the present study we have addressed the effect of a continuous *Plasmodium berghei *infection on the resistance to infection and global gene expression in *Anopheles gambiae*.

Exposure of *A. gambiae *to *P. berghei*-infected blood and infection for 16 generations resulted in a decreased susceptibility to infection, altered constitutive expression levels for approximately 2.4% of the mosquito's total transcriptome and a lower basal level of immune genes expression, including several anti-*Plasmodium *factors. The infection-responsiveness for several defense genes was elevated in the *P. berghei *exposed mosquito colonies.

**Conclusion:**

Our study establishes the existence of a selective pressure exerted by the parasite *P. berghei *on the malaria vector *A. gambiae *that results in a decreased permissiveness to infection and changes in the mosquito transcriptome regulation that suggest a decreased constitutive immune gene activity but a more potent immune response upon *Plasmodium *challenge.

## Background

*Plasmodium*, the causative agent of malaria, exploits the female mosquito's need for human blood as a means of spreading the disease. After the ingestion of an infected blood meal, *Plasmodium *undergoes a complex life cycle in the mosquito that lasts for 14–18 days, leading to the development of infectious sporozoites in the salivary glands. The efficacy of *Plasmodium *infection in the mosquito is usually low but still adequate to permit transmission; the great majority of parasites are eliminated at various stages of development, and the mosquito's innate immune system has been linked with this killing. Thus, the mosquito's innate immune system is a determinant of vector competence. Some studies have documented an impact of *Plasmodium *infection on mosquito physiology and fitness in the wild; under laboratory conditions, the artificially high infection levels that can be achieved are frequently associated with significant mortality at the time when ookinetes invade the midgut epithelium [1]. A variety of mosquito biological processes are also affected by ingestion of infected blood, even in the absence of ookinete invasion of the midgut, suggesting that the specific biochemical composition and various constituents of infected blood are sensed by the mosquito's immune system and present a differential stimulus to the mosquito, when compared to normal non-infected blood [2-4].

*Anopheles *mosquitoes can easily be genetically selected for differential resistance to *Plasmodium*. Two major mechanisms for refractoriness to infection have been described. The first corresponds to a reduction in the number of parasites or their complete elimination during their invasion of the mosquito's midgut cells [5]. Dead ookinetes appear to be surrounded by granular and filamentous material, and they finally break, releasing hemozoin pigment granules into the host cells. The second refractory mechanism occurs when the ookinetes reach the basal lamina; this reaction is responsible for melanotic encapsulation of the humoral type [6]. Several studies have also shown variability in the mosquito's artificial selection in terms of its refractoriness or susceptibility to various *Plasmodium *parasite species and strains [6-15].

Disease resistance traits often have high heritability and high levels of additive genetic variation [16,17]. Several studies have examined the quantitative genetics of immune defense in invertebrates and have found significant levels of additive genetic variation in a range of traits, such as antibacterial activity, phenoloxidase (PO) activity, encapsulation ability, hemocyte density and hemocyte phagocytotic activity [18,19].

The anti-pathogen specificity and efficacy of the insect's innate immune system are shaped by its particular microbial exposure, which can vary significantly between different ecological niches. The magnitude and specificity of the selective pressure imposed by the *Plasmodium *exposure on its mosquito vectors is unclear but has been shown to affect the frequencies of certain immune genes alleles. However, the level of exposure is rather low in the wild, since only a few percent of all mosquitoes are ever infected with *Plasmodium *[20,21].

Gene transcription is intimately related to function and can therefore be used to study the functional responses of an organism to various stimuli, including infection. As is true for gene sequences, gene regulation also responds to selective pressure with adaptations that reflect changes in the expression levels of the genes involved in the physiological systems affected by the imposed parameters.

Here we have used a mass selection approach to assess the selective (evolutionary) impact of exposure to *Plasmodium*-infected blood and infection on mosquito permissiveness to parasite infection and global gene expression. *A. gambiae *mosquitoes were maintained on malaria-infected blood through several generations and then studied with regard to their *Plasmodium *susceptibility and changes in global gene expression patterns, comparing them to those in mosquitoes maintained on normal non-infected blood. Our study shows that continuous exposure to *Plasmodium*-infected blood and infection leads to adaptations that include a decreased permissiveness to infection and a lower basal expression level of anti-*Plasmodium *and other immune-related genes.

## Results and Discussions

### Continuous *Plasmodium *exposure results in a decreased permissiveness to infection

After 16 successive generations of continuously feeding on *P. berghei*-infected mouse blood (exposed *A. gambiae *lines A and B) or normal non-infected mouse blood (non-exposed control line C), we found no significant differences in mortality, size or other visible characteristics between the exposed and control lines. The mortality ranged from 15%–50% (Additional File 1 Table), and death mostly occurred between 24 and 48 h after feeding. However, the permissiveness to *Plasmodium *infection at the pre-mature oocyst stage differentiated substantially between the exposed lines and the control line.

**Figure 1:**
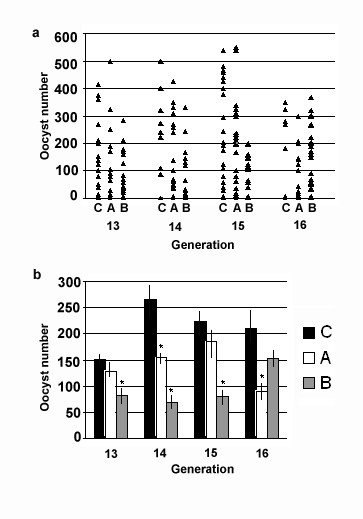
Oocysts numbers in four different generations (13–16) of control (C) and exposed lines (A and B) of *Anopheles gambiae *after infection with *Plasmodium berghei*. **a**: Individual values (each dot represents the oocysts number in the midgut of each mosquito). **b**: Average and standard error of each group of individual values for generations 13–16. Asterisks indicate statistically significant differences (Mann-Whitney test) with respect to control values (*P < 0.05). (see Additional File 3 Table).

After 13 generations, differences at the infection phenotype could already be detected between the continuously exposed and the control lines (Fig. 1a). The exposed lines showed a general tendency toward a lower permissiveness to *Plasmodium*, with a few deviations for some generations and lines (Fig. 1). The levels of infection of generations 13, 14, and 15 of the exposed line B were significantly lower than those of the control line (43%, 74%, and 64%, respectively). Generation 16 displayed only a 27% decrease in infection level (Fig. 1b and Additional File 3 Table). For the same generations (13–16), the exposed line A showed a reduction in the number of oocysts of 10%, 44%, 14%, and 57%, respectively (Fig. 1b and Additional File 3 Table).

The statistically insignificant (27%, 10% and 14%) reduction in *Plasmodium *permissiveness observed for some of the generations of the exposed lines could be due to a number of factors, such as small differences in feeding behavior and the general nutritional and health status of the mosquitoes that can be greatly influenced by rearing conditions such as larval densities. Due to the labor intense nature of rearing several independent mosquito colonies only a limited number (15–40) of mosquitoes were assayed for oocysts number. This could partly also have contributed to this trend of insignificance. However, the exposed lines never displayed a higher permissiveness to *Plasmodium *than that of the control line in any of the assayed generations.

### Divergence in the constitutive transcriptome of *A. gambiae *occurs during continuous exposure to *Plasmodium*

The differences observed in infection phenotype between control and exposed mosquitoes suggest that continuous exposure to *Plasmodium*-infected blood and infection reduces the permissiveness of mosquitoes to *Plasmodium *at the pre-mature oocyst stage. This reduction could be attributed to differences in the transcription levels of genes that can control *Plasmodium *infection. Therefore, we sought to determine the effect of continuous malaria exposure on the global transcriptome of naïve non-blood fed mosquitoes which had been raised on *P. berghei *infected blood (exposed) for thirteen and more consecutive generations. For this purpose, we used microarray analysis to compare mRNA abundance between RNAs from naïve 4-days-old female mosquitoes of the exposed lines (generations 13, 15, and 16 of line A and generations 14 and 15 of line B) and the non-exposed control line (generations 15 and 16) that had been raised on non-infected blood.

**Figure 2:**
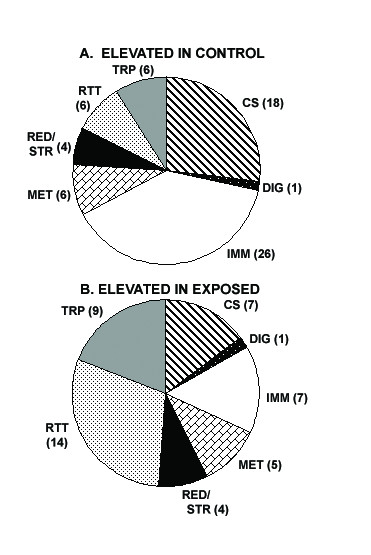
Pie charts showing functional gene class distributions of differentially expressed genes between naïve non-blood fed mosquitoes of the exposed lines (generations 13 to 16) and the non-exposed control lines (generations 15 and 16). The genes are classified in seven different groups according to their predicted functions and shown in two subdivisions, (A) genes that have higher expression levels in the naïve non-blood fed non-exposed control lines and (B) genes that have consistent higher expression levels in the generations 13 to 16 of the exposed lines. [CS: cytoskeletal and structural genes, DIG: digestion-related genes, IMM: immune genes, MET: genes involved in metabolism, RED/STR: redox and stress-related genes, R/T/T: replication/transcription/translation related genes, TRP: transport-related genes]. (see Additional Files 4 and 5 tables).

**Figure 3:**
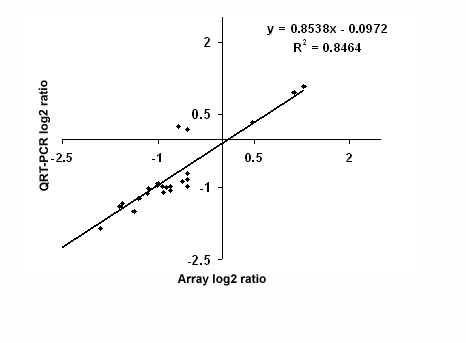
Validation of the microarray data by real-time PCR (qRT-PCR). Log_2_-transformed values from the microarray and qRT-PCR analyses of the TEP1, TEP4, two GSTs, one cytochrome P450, cecropin 3, and apolipoprotein D genes, from naïve mosquitoes of the exposed lines A and B, from generations 13–16 (see Additional File 6 table).

Fig. 2 shows the functional class distribution of those genes that showed significant differential expression between naïve non-blood-fed mosquitoes of the exposed lines (generations 14 and 15 of line B and 13, 15 and 16 of line A) and the control line (pooled generations 15 and 16). In all, continuous exposure to malaria-infected blood and infection caused a significant change in the constitutive transcription level of 2.39% of the total predicted transcriptome (16,148 genes, according to the version 35.2 annotation); 0.83% (124) of these transcripts showed a higher level of expression in the exposed lines, and 1.56% (230) had a higher expression level in the non-exposed control line (Fig. 2, Additional Files 4 and 5 Tables). Among the putative immune genes, only 7 were expressed at higher levels in the exposed lines, while 26 were expressed at higher levels in the non-exposed control line (Fig. 2, Additional Files 4 and 5 Tables). A similar pattern was observed for genes encoding cytoskeletal and structural components, with 7 showing a higher expression in the exposed lines and 18 a higher expression in the control non-exposed line. For the other functional gene groups, no such differences in expression levels were observed; for instance the digestion-related genes group (33 vs 49) and the genes with putative roles in metabolism (5 vs 6). To validate the robustness of the microarray results, we analyzed eight genes from the immunity-, redox/stress-, and transport-related functional groups by qRT-PCR (from Fig. 2, Additional Files 4 and 5 Tables), comparing the expression of these genes between naïve adults of the two exposed lines to the control line. The cDNA templates were normalized to the *A. gambiae *S7 gene, and the -fold differences were calculated as described in Experimental Procedures. These analyses showed a highly significant correlation (Pearson coefficient = 0.91; best-fit linear regression, R^2 ^= 0.84; slope of the regression line, m = 0.85) between the qRT-PCR and the microarray log_2_-transformed values (Fig. 3 and Additional File 6 Table).

The majority of the differentially expressed genes have no known function, and 45 of these showed a higher expression in the exposed lines, while 115 had a higher level of expression in the control line (Additional Files 4 and 5 Tables). This expression pattern is similar to that of putative immune gene group (7 vs 26) (Pearson's Chi-square p = 0.415) and may therefore indicate an infection-related functional bias for many of these unknown genes. In the assays reported here, gene expression was monitored in the entire mosquito, as opposed to specific tissues and cell types that may be more or less affected by and involved in limiting infection. It is likely that some gene expression signatures related to decreased permissiveness to *Plasmodium *infection in the exposed lines are specific for particular tissues and were not identified in our assays because of a dilution effect related to our use of total mosquito RNA. Nevertheless, the assays showed consistent differences in expression of specific functional gene groups that are likely to relate to the observed differences in infection phenotype between the exposed and control lines. Gene expression was also compared between generation 10 and 16 of the control line with the regular insectary colony of the same mosquito strain to test whether the differential gene expression patterns could have resulted from a potential genetic drift of the control line, rather than the exposure to *Plasmodium*. These assays did not identify any significant overlap with the genes that were differentially expressed between the control and the exposed lines (data not shown), and thereby confirm the impact of exposure to *Plasmodium *as the selective pressure responsible for the observed differences.

### Divergence of immune and stress-related gene expression

A notable number of immune genes showed elevated expression in the non-exposed lines (Fig. 2 and Additional File 4 Table). These genes belonged to a variety of functional classes, such as pattern recognition receptors, signal amplification cascade components, and immune signaling pathway components. The pattern recognition receptors group is dominated by thioester-containing protein (TEP) and fibrinogen domain immunolectins (FBN). Interestingly, five of these putative immune proteins, TEP1, FBN9, infection responsive putative short peptide 1 (IRSP1), IRSP2, and Gram-negative bacteria binding protein (GNBPB1) have been shown in RNAi gene-silencing assays to possess anti-*Plasmodium *activity [4,22-24]. TEP1 is involved in phagocytosis of bacteria and anti-*Plasmodium *defense at the stage of ookinete invasion of the mosquito midgut [22,23]. FBN9 belongs to the fibrinogen domain immunolectin gene family, which is represented by at least 61 FBNs in the *A. gambiae *genome [4,25,26]. IRSP1 and IRSP2 are putative short secreted peptides of unknown function [4]. IRSP1 has homology to the salivary gland secreted peptide gVAG [27]. GNBPs are thought to participate in the immune response of *Anopheles *against *Plasmodium *infection [2,28,29] and were first isolated from *Bombyx mori *through their capacity to bind to the surface of Gram-negative bacteria [30]. In *D. melanogaster*, a GNBP has been linked to the activation of the TOLL pathway, together with a peptidoglycan recognition protein (PGRP) [31,32]. The silencing of GNBPB3 and GNBPB4, as well as GNBPB1 in the mosquito, significantly increases the number of oocysts in the midgut after *P. berghei *infection (Dimopoulos lab, unpublished data). The majorities of the other putative immune genes have never been tested for anti-*Plasmodium *activity but belong to functional classes with members that are known to be involved in the defense against *Plasmodium*. For instance, leucine rich repeats (LRRs) are found in a variety of mosquito immune genes, such as the TOLL-like receptors (TLRs) and the anti-*Plasmodium *factors LRIM1, LRRD7, and APL1 [4,24,33,34]. Several *A. gambiae *CLIP domain serine proteases have been shown to be responsive to bacterial and *Plasmodium *infections [4,25,33]. Finally, transferrin also showed a lower level of expression in the exposed lines. Several studies have previously shown that transferrin is induced upon challenge [25,35]; this protein seems to be implicated in limiting bacterial growth by sequestering iron [35].

Putative immune genes that showed higher constitutive expression in the exposed lines than in the control line (Fig. 2, Additional File 5 Table) included TEP15, FBN30, CED6, cecropin 3, PGRPLC1, C type lectin MA1 (CTLMA1), and a TOLL-like precursor. Of these genes only cecropin has been experimentally linked with anti-*Plasmodium *activity [36]. CED-6 is an adaptor molecule that acts in a signal transduction pathway that specifically mediates the recognition and engulfment of apoptotic cells in *C. elegans *[37]. The four-fold higher expression in the naïve adults of the exposed lines suggests that its regulation was strongly affected by the adaptation to *Plasmodium *exposure. The *A. gambiae *antimicrobial peptide cecropin is active against numerous Gram-negative and Gram-positive bacteria, as well as several species of filamentous fungi and yeasts, and when is injected into *Anopheline *mosquitoes previously infected with a variety of *Plasmodium *species, sporogonic development is disrupted by the abortion of the normal development of oocysts [38-40]. The peptidoglycan recognition protein PGRP has been shown to be involved in TOLL and IMD (immunodeficiency) pathway activation in *D. melanogaster *and to be induced after *Plasmodium *infection [33,41]. Two C-type lectins (CTL4 and CTLMA2) have been shown to protect the ookinetes from destruction in the midgut epithelium [24].

Eight redox/stress-related genes showed differential expression in the exposed and non-exposed colonies. Two cytochrome P450 and two gluthatione-S-transferases (GST) were expressed at higher levels in the non-exposed line, and two other cytochrome P450 and two DNAJ protein genes were expressed at higher levels in the exposed lines (Fig. 2, Additional Files 4 and 5 Tables). Cytochrome P450, one of the major enzymes involved in the detoxification of harmful compounds, plays a major role in the mosquito defense system; it has been linked to DDT and pyrethroid resistance in mosquitoes and other insects [42,43]. GSTs are involved in the detoxification of endogenous compounds, such as peroxidates lipids, and in the metabolism of xenobiotics. GSTs have been reported to be differentially expressed in resistant and susceptible strains of malaria [44,45]. Both cytochrome P450 and GSTs have been shown to be regulated after microbial challenge [25].

### Divergence of expression patterns of other functional gene groups

The cytoskeletal and structural component class of genes displayed a general trend toward lower expression in the exposed lines; the major gene families involved included actins and cuticle proteins (Fig. 2, Additional Files 4 and 5 Tables). In addition to serving as a structural component of the exoskeleton, cuticle proteins play a role in wound healing and are also expressed in hemocytes, one of the major immuno-competent cell types in invertebrates [46,47]. Cuticular proteins have also been shown to participate in the non-self recognition of *E. coli *by interacting with its surface [48]. Previous studies have shown infection-responsive regulation of several different cuticle proteins after challenge with either bacteria or *Plasmodium *[4,25]. Actin, microtubules, and other cytoskeletal proteins have been shown to play a major role in the ookinete's traversal of the midgut epithelial cells and the repair of the midgut epithelium after ookinete penetration [49,50].

Two trypsin genes were found to be differentially expressed: Trypsin 1 displayed elevated expression and Trypsin 7 displayed lower expression in the exposed lines (Fig. 2, Additional Files 4 and 5 Tables). Trypsins are known to influence the infectivity of malaria parasites in the mosquito midgut, and for some *Anopheles*-*Plasmodium *species combinations, trypsins can stimulate or inhibit midgut invasion by the ookinetes [51-53]. This difference in gene expression may also be related to the influence of *Plasmodium*-infected blood on mosquito feeding behavior [54]. Among the putative transport-related genes that showed lower expression in the exposed lines was an apolipoprotein D precursor (APOD), the major component of insect lipid transport and a component of the mosquito's immune system and anti-*Plasmodium *defense [55]. Silencing of an APOD has been shown lower the resistance of *Anopheles *to *Plasmodium *infection [4].

Genes in the diverse functional group are associated with a variety of functions, and those belonging to the unknown group did not display significant homology to genes of known function (Additional Files 4 and 5 Tables).

### Divergence of *Plasmodium *infection-responsive transcription patterns

**Figure 4:**
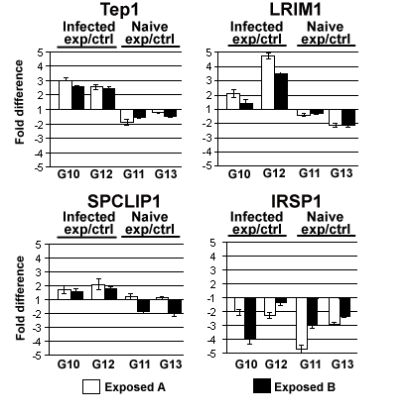
Comparison of expression of the anti-*Plasmodium *immune genes TEP1, *LRIM1*, *SPCLIP1*, and *IRSP1 *between exposed and non-exposed lines upon *P. berghei *infection (Infected) (24 hours after feeding on *P. berghei *infected blood) and at a non-infected non-fed state (Naïve). Expression levels were determined by qRT-PCR in exposed lines A (open box) and B (black box) of generations 10 and 12 (G10 and G12) from infected mosquitoes and generations 11 and 13 (G11 and G13) for the non-infected naïve mosquitoes. The threshold values (Ct) of the four genes were normalized against the *A. gambiae *S7 gene, and the -fold differences in transcript abundance (exposed/non-exposed) were calculated for each gene in the exposed lines A and B with respect to the control line and plotted as bar diagrams. All qRT-PCRs were repeated at least three times, and the mean values are shown in the graph with standard errors indicated (see Additional File 7 Table).

The fact that significantly larger number of immune genes, including anti-*Plasmodium *factors, was expressed at elevated levels in the non-exposed line suggests that continuous *Plasmodium *exposure may also lead to a divergence in the infection-responsive regulation of these genes. Therefore, we compared the expression patterns of four anti-*Plasmodium berghei *factors, TEP1, LRIM1, SPCLIP1 and IRSP1, between the exposed and non-exposed lines in response to *P. berghei *infection at 24 h after feeding (infected), the time at which the ookinetes invade the midgut epithelium [4,23,24]. A comparison was also done for naïve non-blood fed mosquitoes for the same genes. Due to the limited number of available mosquitoes in each generation, these assays used infected mosquitoes from different but consecutive generations to those used for the microarray expression analyses of naïve mosquitoes. Mosquitoes were collected from generations 11 and 13 for assays on naïve expression levels and generations 10 and 12 for evaluation of infection-responsive expression (Fig. 4 and Additional File 7 Table). The constitutive expression levels of the immune genes TEP1, LRIM1, and SPCLIP1 were in most cases higher in the naïve mosquitoes of the non-exposed line than those of the exposed lines (Fig. 4). Infection with *Plasmodium *caused a stronger induction of TEP1, LRIM1, and SPCLIP1 in the exposed lines than in the non-exposed control line: an increase of more than two-fold for TEP1, four-fold for LRIM1, and close to two-fold for SPCLIP1. IRSP1 did not follow the same pattern and displayed lower expression levels in both non-exposed and exposed mosquito lines.

## Conclusion

In the present study, we have shown that continuous exposure to *P. berghei*-infected blood and infection exerts a directional selective pressure upon the mosquito *A. gambiae *that results in a reduced permissiveness to infection by the parasite. This selective pressure also shapes the mosquito's transcriptome expression to produce an unexpected pattern in which the basal transcript levels of innate immune genes, which play a role in anti-*Plasmodium *defense, are decreased. A similar pattern was observed for several other cytoskeletal, structural, and transporter genes, some of which have been linked with *Plasmodium *development in the mosquito (Fig. 2). In contrast, the immune-responsive expression levels of several anti-*Plasmodium *genes were significantly higher in the exposed mosquito lines (Fig. 4).

Pathogens exert a selective pressure on their hosts that can eventually lead to the development of resistance. The course of such selection is dependent on the extent of the genetic variation in the traits that influence host defense, as well as the trade-offs between these traits and other factors that can influence host fitness. In the case of *Plasmodium *infection in *Anopheles*, the exposure to the parasite has been shown to reduce the mosquito's fecundity and longevity [56-58]. A recent study has also showed that there is a significant fitness cost of *P. berghei *infection of *A. gambiae *[59]. The destruction of the parasites in *Anopheles *refractory strains has also been associated with a fitness cost [60,61].

The results of the present study suggest that the mosquitoes' permissiveness to infection is likely to decrease to the point at which higher levels of resistance would have a higher fitness cost than would the tolerance of a certain level of infection. The survival of mosquitoes in each round of infection is likely to be attributable to a trade-off, or rebalancing; an adjustment in the constitutive and infection-responsive levels of gene expression that favors an efficient defense against the parasite without causing an overall decrease in fitness.

The insect innate immune system consists of several independent but functionally overlapping branches that fight pathogens. A genetically mediated enhancement of a specific defense mechanism may therefore render the functional necessity of other defenses less important and thereby lead to their gradual suppression. Our continuous infection assay is likely to have selected for a resistance mechanism that is not reflected at the transcript abundance level, such as an allele polymorphism or a post-transcriptional process that modulates active protein levels. Through its potential activity against the mosquito's constitutive microbial flora, this elevated resistance may, in turn, have resulted in a general down-regulation of other immune genes, including anti-*Plasmodium *factors, that are also active in defending against bacterial infections [4]. A recent study has suggested that the mosquito's anti-*Plasmodium *defense can be mainly attributed to the basal (constitutive) expression of certain anti-*Plasmodium *genes, rather than the responsiveness of these lines to infection, the responsiveness only serving to replenish immune factors during and after infection [62]. According to this model, a mechanism that would result in greater immune protein stability could explain our observation of 1) a lower basal expression level, reflecting a slower protein turnover rate; and 2) an enhanced induction in response to infection, which replenishes the immune proteins that are secreted upon infection. Alternatively, the lower basal expression of immune genes in the exposed lines may have evolved to enable a more potent response upon *Plasmodium *infection. Upon a stress condition, such as *Plasmodium *infection, the mosquito needs to focus a large proportion of its resources on the defense. Hence, a high constitutive expression of a broad range of immune genes would consume resources needed for rapid and strong activation of a more specific repertoire of genes. According to this hypothesis, consecutive *Plasmodium *infections may select for mosquitoes with a lower constitutive consumption of resources because of their ability to more efficiently relocate resources for immune response.

It should be noted that the laboratory model used in this study may represent an extreme situation that does not occur in nature. *P. berghei *achieves unnaturally high infections in *A. gambiae*, and our assays were designed to expose all mosquitoes of each consecutive generation to *Plasmodium*-infected blood and infection. In contrast, the prevalence of *P. falciparum *infection in the field rarely exceeds 20%, and the infection levels rarely exceed 2–3 oocysts per infected mosquito [20]. Furthermore, the parasite's ability to develop the capacity to evade the mosquito's immune response was not reflected in this study, since the parasites were derived from the same stock for each infection assay. However, this model still provides interesting insights into the changes in susceptibility and transcriptional regulation that may be associated with *Plasmodium *infection, and it establishes a link between the mosquito's immune and stress response systems and adaptation-driven resistance to *Plasmodium*.

## Methods

### Mosquito rearing and *Plasmodium *infections

Three independent lines (two exposed or experimental, A and B, and one non-exposed or control, C) of the *A. gambiae *Keele strain (~400 adults per line) were maintained at identical conditions on sugar solution at 27°C and 70% humidity with a 12-h light/dark cycle, according to standard rearing procedures [63]. The exposed lines A and B were reared on GFP *P. berghei*-infected Swiss Webster mice [64] for 16 consecutive generations; the control line was reared on uninfected Swiss Webster mice for the same number of generations. *Plasmodium *infections were performed according to [29]. Briefly, GFP *P*. berghei parasites were passaged in Swiss Webster mice, and parasitemia was determined by Giemsa-stained blood films. The exflagellation events were controlled at averagely 2 to 3 per 20× field. For each feeding experiment, the two exposed lines were fed on the same infected mouse/mice. When two mice were required for one experiment, mice with similar infection levels were used and feedings were done by placing the mice with head part on one cage and tail part on another cage. Every 5 minutes, the positions were flipped to ensure ingestion of equal parasite numbers. The fed mosquitoes from either exposed lines or the control line were firstly kept at 19°C for about 2 days before transferred back to normal conditioned insectary. For colony maintenance, the unfed female mosquitoes from either exposed lines or the control line were removed from the cage after blood feeding, and only fully engorged females were used for eggs laying to produce the next generation of progeny. The larvae hatched ~2–3 days after eggs laying, and the pupae were collected ~10–12 days later; they matured to adult mosquitoes after an additional ~1–2 days. The mosquitoes were maintained on sugar solution, and 5- to 6-day-old mosquitoes were blood-fed (with infected or uninfected blood) and reared in this manner for 16 generations.

For analyses related to mortality, infection phenotype (oocysts number in the midgut), and the expression of immune genes after infection, a pool of mosquitoes from the control line (~80 adults) from each generation was also fed on the same infected mice as were the two exposed lines. Two mice with similar infection levels were laid on the cages with head/tail on two different cages, and the positions of the mice were rotated every 2 to 5 minutes between the cages. The mosquitoes that had fed on infected and naïve blood were firstly kept at 19°C [65] for about 2 days before transferred back to normal conditioned insectary, and their mortality was determined 3–4 days after feeding (Additional File 1 Table). The transfer of mosquitoes to 27°C at two days after feeding did not affect *P. berghei *development as indicated by the formation of mature oocysts at 9 days after feeding. The majority of mosquito mortality occurred between 24 to 48 hours after feeding on infected blood as previously described [1]. Control line fed on the non-infected blood showed no significant mortality. After 7–9 days, midguts were dissected (~15–20) to determine the level of infection, as measured by oocysts numbers (Fig. 1 and Additional File 3 Table). For RNA isolation, adult females (~20 per line) from all three lines were also collected in each generation before feeding on either infected or non-infected blood (naïve) and after feeding on infected blood (infected). All the assays were done in triplicate.

### RNA extraction and quantitative real-time PCR (QRT-PCR)

RNA was extracted from whole mosquitoes using the RNeasy kit (QIAGEN). Quantification of RNA was performed using a Biophotometer spectrophotometer. RNA samples were treated with Turbo DNase and reverse-transcribed using Superscript II (Invitrogen) with oligo dT_20_. Real-time quantification was performed using the QuantiTect SYBR Green PCR Kit (Qiagen) and ABI Detection System ABI Prism 7300 (Applied Biosystems, California, USA). All PCR reactions were performed in triplicate. The specificity of the PCR reactions was assessed by analysis of the melting curves for each data point. The ribosomal protein S7 gene was used for normalization of the cDNA templates and to calculate the threshold values (Ct). The -fold differences in expression level were calculated by the standard E^ΔΔCt ^[66] method. Primers used for all qRT-PCR (Fig. 3 and Fig. 4) are listed in Additional File 2 Table.

### Microarray analysis

The Low RNA Input Fluorescent Linear Amplification Kit from Agilent Technologies was used to synthesize Cy-3 and Cy-5 labeled samples from 2 microgram RNA according to the manufacturer's instructions. RNA from the control line generations 15 and 16 were pooled and used as one control sample (labeled with Cy-3-dUTP fluorescent nucleotides) to hybridize to the microarray against the five exposed lines samples (exposed line A from generations 13, 15, and 16, and exposed line B from generations 14 and 15) and labeled with Cy-5-dUTP fluorescent nucleotides. Microarray hybridizations were performed as previously described [4], and all assays were done in triplicate. Spot intensities were measured with a GenePix 7000 autoloader scanner (Axon Instruments) using a 10 μm pixel size. The laser power was set to 60%, and the PMT was adjusted to maximize the effective dynamic range and minimize pixel saturation. Images were inspected manually using GenePix Pro 6.0 software (Axon Instruments, Union City, CA), and any spots that were covered with hybridization artifacts were removed and not included in further analysis. Express Converter software was used to convert the Gene Pix results files to MEV files and be analyzed by using TIGR-MIDAS software, which is available online from TIGR [67]. The data sets were filtered using a signal cut-off intensity of 100 to remove low-intensity/poorly hybridized spots from the analysis. Loc-Fit normalization (LOWESS) was performed independently for each data set. The TMEV software, available online [67], was finally used for SAM analysis with a 5% false discovery rate (FDR). Only transcripts that had signal values above the log_2 _cutoff values of ± 0.807 were used for further analysis as previously described [4,68] (Fig. 2, Additional Files 4, and 5 Tables).

## List of Abbreviations

APL1: *Anopheles Plasmodium*-responsive leucine-rich repeat 1

APOD: apolipoprotein D precursor

CED6: cell death abnormality 6

CS: cytoskeletal and structural genes

CTL: C type lectin

DDT: dichloro-diphenyl-trichloroethane

DIG: digestion-related genes

FBN: fibrinogen domain immunolectin

FDR: false discovery rate

GFP: green fluorescent protein

GNBP: Gram-negative bacteria binding protein

GST: Gluthatione-S-transferase

IMM: immune genes

IRSP: infection responsive putative short peptide

LRIM1: leucine-rich repeat immune protein

LRR: leucine rich repeat

MET: genes involved in metabolism

PGRP: peptidoglycan recognition protein

PMT: photomultiplier tube

PO: phenoloxidase

qRT-PCR: quantitative real-time PCR

R/T/T: replication/transcription/translation related genes

RED/STR: redox and stress-related genes

SAM: Significance analysis of microarrays

SPCLIP1: serine protease CLIP1

TEP: thioester-containing protein

TLR: TOLL-like receptor

TRP: transport-related genes

## Authors' contributions

RA, SD and YD contributed equally towards the experimental design, biological assays, data analyses, manuscript writing and GD contributed with experimental design, data analyses and writing the manuscript. All authors read and approved the final manuscript.

## Supplementary Material

Additional file 1 Mortality of *P. berghei *infected mosquitoes of the exposed and non-exposed control lines at 3–4 days after infected blood feeding. Mortality of *P. berghei *infected mosquitoes of the exposed and non-exposed control lines (from generations 7 to 14) at 3–4 days after infected blood feeding are indicated in the table and graph. Click here for file

Additional file 3 Oocyst numbers in midguts of infected blood-fed mosquitoes from generations 13–16. Numbers of oocysts in control and exposed lines (A and B), of generations 13–16 on day 7 after feeding on *P. berghei*-infected mice. Total midgut dissected (midguts #), mean and standard error of oocysts numbers (mean ± SE), and p-values from the Mann-Whitney test are presented. Zero oocysts are also included for the calculation of mean oocysts numbers. NS = not significant; S = significant difference. Click here for file

Additional file 4 Genes with higher expression in the control line. Genes with higher expression in the control line as compared to the exposed line. Gene names, their corresponding transcript IDs (ENSANGT numbers), and the average log_2_-fold differences are shown (for Gen 13A, Gen 14B, Gen15A, Gen15B, and Gen16A). The fold difference represents the average of the five exposed sets of mosquitoes. The genes are sorted into groups (column C) according to their function (see also Fig. 2 for nomenclature of the group names). CS = cytoskeleton, structure; D = diverse function; DIG = digestive; I = immunity; M = metabolism; RSM = redox, stress, mitochondrial; RTT = replication, transcription, translation; TRP = transport; U = unknown function; D = diverse function. The nomenclature used for the cytochrome P450 and GSTs follows [42]. Click here for file

Additional file 5 Genes with higher expression in the exposed lines. Genes with higher expression in the exposed lines as compared to the control line. Gene names, their corresponding transcript IDs (ENSANGT numbers), and the average log_2_-fold differences are shown (Gen 13A, Gen 14B, Gen15A, Gen15B, and Gen16A). The fold difference represents the average of the five exposed sets of mosquitoes. The genes are sorted into groups (column C) according to their function (see also Fig. 2 for nomenclature of the group names). CS = cytoskeleton, structure; D = diverse function; DIG = digestive; I = immunity; M = metabolism; RSM = redox, stress, mitochondrial; RTT = replication, transcription, translation; TRP = transport; U = unknown function; D = diverse function. The nomenclature used for the cytochrome P450 follows [42]. Click here for file

Additional file 6 Correlation of microarray expression data with real-time qRT-PCR expression data. The log _2 _ratios (exposed/control) of the gene expression from both the microarray and qRT-PCR analyses are presented in the table. The gene name, generation number, and selection line name are listed in column 1. The transcript ID is listed in column 2, with the portion "ENSANGT00000" removed from the ENSEMBL transcript ID. Click here for file

Additional file 7 Real-time quantitative PCR (qRT-PCR) based expression analyses of immune genes in naïve and infected blood-fed mosquitoes (see Fig. 4). This table provides raw data for assays presented in Fig. 4. qRT-PCR expression values for four immune genes (Tep1, LRIM1, SPCLIP1, and IRSP1) from generations 10–13. ^a ^fold = fold changes of exposed/control (if up-regulated) and control/exposed (if down regulated [25]); ^b ^S.E. = standard error of three replicates. The transcript IDs of the immune genes are also shown; the ENSANGT00000 portion is shown as E. Click here for file

Additional file 2 Primer sets used for the qRT-PCR in Fig. 3 and Fig. 4. Primer sets used for the qRT-PCR in Fig. 3 and Fig. 4. Click here for file
