# Supplementary material for: Continuous exposure to Plasmodium results in decreased susceptibility and transcriptomic divergence of the Anopheles gambiae immune system
Source: BMC Genomics. 2007 Dec 5;8:451. doi: 10.1186/1471-2164-8-451 (PMC2234432; doi:10.1186/1471-2164-8-451)

**Additional File 1.** Mortality of *P. berghei* infected mosquitoes of the exposed and non-exposed control lines (from generations 7 to 14) at 3-4 days after infected blood feeding are indicated in the table and graph.

| Generation number | Control  (non exposed) | Exposed line A | Exposed line B |
| --- | --- | --- | --- |
| Gen 7 | 13 | 15 | 13 |
| Gen 8 | 71 | 57 | 64 |
| Gen 9 | 24 | 34 | 47 |
| Gen 10 | 39 | 44 | 52 |
| Gen 11 | 29 | 43 | 22 |
| Gen 12 | 8 | 46 | 25 |
| Gen 13 | 27 | 24 | 18 |
| Gen 14 | 51 | 60 | 62 |


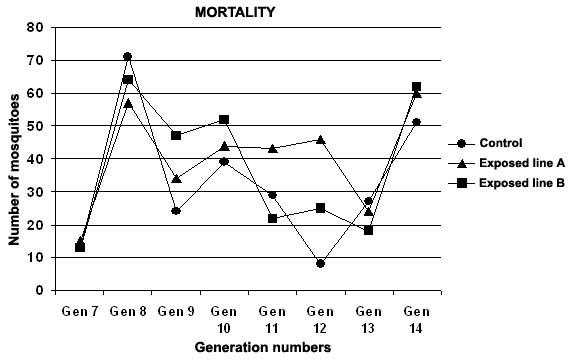

Supplement: Additional file 1 — Mortality of P. berghei infected mosquitoes of the exposed and non-exposed control lines at 3–4 days after infected blood feeding. Mortality of P. berghei infected mosquitoes of the exposed and non-exposed control lines (from generations 7 to 14) at 3–4 days after infected blood feeding are indicated in the table and graph. [file 1471-2164-8-451-S1.doc]
