# Supplementary material for: Continuous exposure to Plasmodium results in decreased susceptibility and transcriptomic divergence of the Anopheles gambiae immune system
Source: BMC Genomics. 2007 Dec 5;8:451. doi: 10.1186/1471-2164-8-451 (PMC2234432; doi:10.1186/1471-2164-8-451)
Supplement: Additional file 3 — Oocyst numbers in midguts of infected blood-fed mosquitoes from generations 13–16. Numbers of oocysts in control and exposed lines (A and B), of generations 13–16 on day 7 after feeding on P. berghei-infected mice. Total midgut dissected (midguts #), mean and standard error of oocysts numbers (mean ± SE), and p-values from the Mann-Whitney test are presented. Zero oocysts are also included for the calculation of mean oocysts numbers. NS = not significant; S = significant difference. [file 1471-2164-8-451-S3.doc]

**Additional File 3.** Oocyst numbers in midguts of infected blood-fed mosquitoes from generations 13-16.

| Generation  # | Type | Midguts # | Mean oocysts  ± SE | p value (Mann Whitney test) | Significance |
| --- | --- | --- | --- | --- | --- |
| Gen 13 | Control | 15 | 150.1 ± 10.7 | - | - |
|  | Exp line A | 15 | 127.4 ± 24.03 | 0.410 | NS |
|  | Exp line B | 15 | 81.8 ± 17.25 | 0.038 | S |
| Gen 14 | Control | 13 | 255.2 ± 30.5 | - | - |
|  | Exp line A | 13 | 152.3 ± 16.02 | 0.037 | S |
|  | Exp line B | 13 | 65.8 ± 20.12 | 0.001 | S |
| Gen 15 | Control | 20 | 221.2 ± 17.2 | - | - |
|  | Exp line A | 20 | 180.3 ± 25.3 | 0.310 | NS |
|  | Exp line B | 20 | 80.5 ± 10.3 | 0.001 | S |
| Gen 16 | Control | 12 | 208.2 ± 38.9 | - | - |
|  | Exp line A | 12 | 91.3 ± 13.2 | 0.001 | NS |
|  | Exp line B | 12 | 151.1 ± 15.6 | 0.239 | S |

Numbers of oocysts in control and exposed lines (A and B), of generations 13-16 on day 7 after feeding on *P. berghei*-infected mice. Total midgut dissected (midguts #), mean and standard error of oocysts numbers (mean ± SE), and p-values from the Mann-Whitney test are presented. Zero oocysts are also included for the calculation of mean oocysts numbers. NS=not significant; S=significant difference.
