# Supplementary material for: Continuous exposure to Plasmodium results in decreased susceptibility and transcriptomic divergence of the Anopheles gambiae immune system
Source: BMC Genomics. 2007 Dec 5;8:451. doi: 10.1186/1471-2164-8-451 (PMC2234432; doi:10.1186/1471-2164-8-451)
Supplement: Additional file 6 — Correlation of microarray expression data with real-time qRT-PCR expression data. The log 2 ratios (exposed/control) of the gene expression from both the microarray and qRT-PCR analyses are presented in the table. The gene name, generation number, and selection line name are listed in column 1. The transcript ID is listed in column 2, with the portion "ENSANGT00000" removed from the ENSEMBL transcript ID. [file 1471-2164-8-451-S6.doc]

**Additional File 6**. Correlation of microarray expression data with real-time QRT-PCR expression data.

| Gene (gene #,type) | Transcript ID | QRT -PCR  (log_2_ ratio) | Microarray  (log_2_ ratio) |
| --- | --- | --- | --- |
| Tep 1 (gen 15, Exp A) | **E021282** | -1.13 | -1.17 |
| Tep 1 (gen 16, Exp A) | **E021282** | -1.34 | -1.56 |
| Tep 1 (gen 15, Exp B) | **E021282** | -0.83 | -0.54 |
| Tep 4 (gen 15, Exp A) | E021216 | -1.04 | -1.16 |
| Tep 4 (gen 16, Exp A) | E021216 | -1.07 | -0.80 |
| Tep 4 (gen 15, Exp B) | E021216 | -0.99 | -0.81 |
| GST S1-1 (gen 15, Exp A) | E023810 | -1.98 | -2.62 |
| GST S1-1 (gen 16, Exp A) | E023810 | -1.84 | -1.90 |
| GST S1-1 (gen 15, Exp B) | E023810 | -0.99 | -0.94 |
| GST S1-2 (gen 15, Exp A) | E010247 | -1.50 | -1.38 |
| GST S1-2 (gen 16, Exp A) | E010247 | -0.91 | -1.00 |
| GST S1-2 (gen 15, Exp B) | E010247 | -1.01 | -0.87 |
| Cyt P450 (gen 15, Exp A) | E016967 | -0.97 | -1.01 |
| Cyt P450 (gen 16, Exp A) | E016967 | -1.23 | -1.30 |
| Cyt P450 (gen 15, Exp B) | E016967 | -1.10 | -0.91 |
| IRSP1 (gen 14, Exp B) | E021028 | -0.71 | -0.54 |
| IRSP1 (gen 15, Exp A) | E021028 | -0.88 | -0.63 |
| IRSP1 (gen 15, Exp B) | E021028 | -1.40 | -1.60 |
| Cecropin 3 (gen 13, Exp A) | E011995 | 0.97 | 1.12 |
| Cecropin 3 (gen 14, Exp B) | E011995 | 1.07 | 1.28 |
| Cecropin 3 (gen 15, Exp A) | E011995 | 0.34 | 0.47 |
| APOD (gen 14, Exp B) | E028106 | 0.25 | -0.68 |
| APOD (gen 15, Exp A) | E028106 | -0.99 | -0.54 |
| APOD (gen 16, Exp A) | E028106 | 0.18 | -0.55 |

The log _2_ ratios (exposed /control) of the gene expression from both the microarray and QRT-PCR analyses (Fig. 2 and Fig. 3 are shown. The gene name, generation number, and selection line name are listed in column 1. The transcript ID is listed in column 2, with the portion “E**NSANGT00000” removed from the ENSEMBL transcript ID.**
