# Supplementary material for: Continuous exposure to Plasmodium results in decreased susceptibility and transcriptomic divergence of the Anopheles gambiae immune system
Source: BMC Genomics. 2007 Dec 5;8:451. doi: 10.1186/1471-2164-8-451 (PMC2234432; doi:10.1186/1471-2164-8-451)
Supplement: Additional file 7 — Real-time quantitative PCR (qRT-PCR) based expression analyses of immune genes in naïve and infected blood-fed mosquitoes (see Fig. 4). This table provides raw data for assays presented in Fig. 4. qRT-PCR expression values for four immune genes (Tep1, LRIM1, SPCLIP1, and IRSP1) from generations 10–13. a fold = fold changes of exposed/control (if up-regulated) and control/exposed (if down regulated [25]); b S.E. = standard error of three replicates. The transcript IDs of the immune genes are also shown; the ENSANGT00000 portion is shown as E. [file 1471-2164-8-451-S7.doc]

**Additional File 7**. Real-time quantitative PCR (QRT-PCR) analyses of immune genes in naïve and infected blood-fed mosquitoes.

| Gene name | Transcript ID | Generation # | Type | Fold ^a^ | S.E. ^b^ |
| --- | --- | --- | --- | --- | --- |
| Tep 1 | **E021282** | Gen 10 | Exp line A | 2.99 | 0.23 |
|  |  |  | Exp line B | 2.54 | 0.03 |
|  |  | Gen 12 | Exp line A | 2.55 | 0.14 |
|  |  |  | Exp line B | 2.5 | 0.22 |
|  |  | Gen 11 | Exp line A | 1.89 {-} | 0.18 |
|  |  |  | Exp line B | 1.54 {-} | 0.04 |
|  |  | Gen 13 | Exp line A | 1.20 {-} | 0.03 |
|  |  |  | Exp line B | 1.45 {-} | 0.04 |
| LRIM 1 | E**013041** | Gen 10 | Exp line A | 2.12 | 0.37 |
|  |  |  | Exp line B | 1.41 | 0.25 |
|  |  | Gen 12 | Exp line A | 4.76 | 0.20 |
|  |  |  | Exp line B | 3.5 | 0.25 |
|  |  | Gen 11 | Exp line A | 1.43 {-} | 0.05 |
|  |  |  | Exp line B | 1.25 {-} | 0.02 |
|  |  | Gen 13 | Exp line A | 2.13 {-} | 0.80 |
|  |  |  | Exp line B | 2.13 {-} | 0.60 |
| SPCLIP1 | E020158 | Gen 10 | Exp line A | 1.7 | 0.32 |
|  |  |  | Exp line B | 1.6 | 0.25 |
|  |  | Gen 12 | Exp line A | 2.07 | 0.53 |
|  |  |  | Exp line B | 1.78 | 0.22 |
|  |  | Gen 11 | Exp line A | 1.03 | 0.09 |
|  |  |  | Exp line B | 1.85 {-} | 0.04 |
|  |  | Gen 13 | Exp line A | 1.11 | 0.04 |
|  |  |  | Exp line B | 2.0 {-} | 0.24 |
| IRSP1 | E021028 | Gen 10 | Exp line A | 2.08 {-} | 0.34 |
|  |  |  | Exp line B | 4.0 {-} | 0.31 |
|  |  | Gen 12 | Exp line A | 2.33 {-} | 0.28 |
|  |  |  | Exp line B | 1.47 {-} | 0.19 |
|  |  | Gen 11 | Exp line A | 4.76 {-} | 0.31 |
|  |  |  | Exp line B | 3.03 {-} | 0.28 |
|  |  | Gen 13 | Exp line A | 2.94 {-} | 0.06 |
|  |  |  | Exp line B | 2.44 {-} | 0.02 |

QRT-PCR values for four immune genes (Tep1, LRIM1, SPCLIP1, and IRSP1) from generations 10-13. ^a^ fold = fold changes of exposed / control (if up-regulated) and control /exposed (if down regulated {-}); ^b^ S.E.=standard error of three replicates. The transcript IDs of the immune genes are also shown; the **ENSANGT00000** portion **is shown as E.**
