# Supplementary material for: Continuous exposure to Plasmodium results in decreased susceptibility and transcriptomic divergence of the Anopheles gambiae immune system
Source: BMC Genomics. 2007 Dec 5;8:451. doi: 10.1186/1471-2164-8-451 (PMC2234432; doi:10.1186/1471-2164-8-451)
Supplement: Additional file 2 — Primer sets used for the qRT-PCR in Fig. 3 and Fig. 4. Primer sets used for the qRT-PCR in Fig. 3 and Fig. 4. [file 1471-2164-8-451-S2.doc]

**Additional File 2. Primer sets used for the RT-qPCR in Fig. 3 and Fig.4**

| Gene/Transcript ID | Primer name | Sequence |
| --- | --- | --- |
| ENSANGG00000009506**ENSANGT00000011995** | Cecropin 3: Forward  Reverse | 5’- GTTTCGGGCGGCTAAGAA-3’OH  5’- AACAAACACGCACGCACA-3’OH |
| **ENSANGG00000007758**  **ENSANGT00000010247** | GSTS1-2: Forward  Reverse | 5’-TGAACAACGAGGTCATTCCA-3’OH  5’- TTGCGACCAGATCAGACTTG-3’OH |
| **ENSANGG00000007758**  **ENSANGT00000023810** | GSTS1-1: Forward  Reverse | 5’- CGACCTGGGCTGATGTTTAT-3’OH  5’- GTTGCTCTTCTCGGCAAATC-3’OH |
| **ENSANGG00000014478**  **ENSANGT00000016967** | Cyt P450: Forward  (CYP6P2) Reverse | 5’- TGGGATTGATTACGCTGCT -3’OH  5’- AGCGATGCTTTGTTTTGCT - 3’OH |
| **ENSANGG00000010552**  **ENSANGT00000013041** | LRIM: Forward  Reverse | 5’- ACGTTAAGGGACAGGTCGT -3’OH  5’-AGTGAAACCCGTTGCCGCGC-3’OH |
| **ENSANGG00000018727**  **ENSANGT00000021216** | Tep4: Forward  Reverse | 5’- TGTTGATTGCACTGCTGGAG-3’OH  5’- TCCCGTCCACGTACTTCTCC-3’OH |
| ENSANGG00000018793**ENSANGT00000021282** | Tep1: Forward Reverse | 5’- ACATGGCACTGATTGAGGT-3’OH  5’- TCGCACAAATTCTGCTTGT-3’OH |
| ENSANGG00000017669ENSANGT00000020158 | SPCLIP1: Forward  Reverse | 5’- GTCACCGAACACGTCCAAC-3’OH  5’- CTGCATGGCCCTACGTCTA-3’OH |
| ENSANGG00000018539ENSANGT00000021028 | IRSP1: Forward  Reverse | 5’- CTGGAAGGATGGACAGATG-3’OH  5’- GGATTGAAACGGATCAAGG-3’OH |
| **ENSANGG00000023945 ENSANGT00000028106** | APOD: Forward  Reverse | 5’- CACCGAAAACCTCCAACTA - 3’OH  5’- TTGCATTTTGCCTGACTTTG -3’OH |
